# KLF8 is associated with poor prognosis and regulates glycolysis by targeting GLUT4 in gastric cancer

**DOI:** 10.1111/jcmm.14378

**Published:** 2019-05-24

**Authors:** Anwei Mao, Xiang Zhou, Yanxia Liu, Junbin Ding, Aiyu Miao, Gaofeng Pan

**Affiliations:** ^1^ Department of General Surgery Minhang Hospital, Fudan University Minhang, Shanghai China; ^2^ Department of Nursing Minhang Hospital, Fudan University Minhang, Shanghai China; ^3^ Department of Ultrasound Fudan University Shanghai Cancer Center Shanghai China; ^4^ Department of Oncology Shanghai Medical College, Fudan University Shanghai China

**Keywords:** gastric cancer, KLF8, prognosis, Warburg effect

## Abstract

Krüppel‐like transcription factor (KLF) family is involved in tumorigenesis in different types of cancer. However, the importance of KLF family in gastric cancer is unclear. Here, we examined KLF gene expression in five paired liver metastases and primary gastric cancer tissues by RT‐PCR, and immunohistochemistry was used to study KLF8 expression in 206 gastric cancer samples. The impact of KLF8 expression on glycolysis, an altered energy metabolism that characterizes cancer cells, was evaluated. KLF8 showed the highest up‐regulation in liver metastases compared with primary tumours among all KLF members. Higher KLF8 expression associated with larger tumour size (*P* < 0.001), advanced T stage (*P* = 0.003) and N stage (*P* < 0.001). High KLF8 expression implied shorter survival outcome in both TCGA and validation cohort (*P* < 0.05). Silencing KLF8 expression impaired the glycolysis rate of gastric cancer cells in vitro. Moreover, high KLF8 expression positively associated with SUVmax in patient samples. KLF8 activated the GLUT4 promoter activity in a dose‐dependent manner (*P* < 0.05). Importantly, KLF8 and GLUT4 showed consistent expression patterns in gastric cancer tissues. These findings suggest that KLF8 modulates glycolysis by targeting GLUT4 and could serve as a novel biomarker for survival and potential therapeutic target in gastric cancer.

## 1 INTRODUCTION

Gastric cancer is the fourth most common cancer in the world^1^ and the second most common cancer in China.^2^ Despite recent advances in surgery, chemotherapy and targeted therapy, the prognosis of patients with gastric cancer remains dismal. Nearly half of gastric cancer patients will suffer tumour recurrence after radical resection.^3^, ^4^, ^5^ Local recurrences and distant metastases are the main factors associated with death in gastric cancer patients.^6^ Therefore, better understanding of the mechanisms underlying tumour progression and metastasis in gastric cancer will lead to the identification of potential prognostic factors and potential targets for therapeutic treatments.

The Krüppel‐like transcription factor (KLF) family consists of 17 distinct members involved in the regulation of different cellular physiological processes,^7^, ^8^ including proliferation, apoptosis, differentiation and migration.^9^, ^10^, ^11^, ^12^ KLF8 is a relatively new member of the KLF family and is a sequence‐specific DNA‐binding protein that recognizes the CACCC‐box element.^13^, ^14^ Recent studies demonstrated that KLF8 induces epithelial‐to‐mesenchymal transition (EMT), stem cell characteristics and drug resistance in various cancers.^15^, ^16^, ^17^, ^18^, ^19^, ^20^ KLF8 acts as a dual transcriptional factor that can either repress or activate transcription of target genes including β‐catenin,^18^ CXCR4^19^ and E‐cadherin genes.^20^ KLF8 is overexpressed in gastric cancer, and ectopic KLF8 plays vital roles in various pathological processes. Silencing KLF8 expression significantly attenuated gastric cancer cell proliferation^21^, ^22^ and invasion.^22^ KLF8 is also an important regulator of TGF‐beta‐17 or hypoxia‐23 induced EMT and an inducer of hypoxia‐dependent multidrug resistance^13^ and angiogenesis.^24^ High KLF8 expression is generally regarded as a negative predictor of survival for gastric cancer.^24^, ^25^ However, whether KLF8 plays other functions in gastric cancer has not been investigated.

Metabolic reprogramming plays critical roles in tumorigenesis and metastasis and is a fundamental characteristic of cancer cells.^26^, ^27^ Differentiated cells normally rely primarily on the oxidation of pyruvate in the mitochondria to generate energy for cellular activities. However, rapidly growing cancer cells rely on aerobic glycolysis to generate energy, a phenomenon termed the Warburg effect.^28^ The Warburg effect not only provides cancer cells with ATP and nutrients but also creates an acidic environment that leads to destruction of extracellular matrix and facilitates metastasis.^26^, ^29^ Therefore, identifying key players that regulate glycolysis may contribute to the development of diagnostic and treatment strategies for cancer, including colorectal cancer.

Here, we performed a systematic study of KLF family expression in hepatic metastases and matched primary tumours by RT‐PCR and found that KLF8 was significantly up‐regulated in liver metastases. We investigated KLF8 expression and function in gastric cancer and its clinical significance. Our results show that KLF8 can promote glycolysis in gastric cancer by targeting GLUT4.

## 2 MATERIALS AND METHODS

### 2.1 Patients and samples

Three patient cohorts were used to explore the clinical importance of KLF8 expression in gastric cancer. In one group, tissues from five patients with liver metastases and who received simultaneous primary and metastases tumour resection were used for primary screening of KLF genes involved in metastases. Tissues were kept in RNA later and stored at −20ºC immediately after resection. The second cohort consisted of 206 patients with pathologically diagnosed gastric adenocarcinoma. All patients underwent radical gastrectomy and received standard adjunctive therapy after surgery. Samples were analysed by immunohistochemistry (IHC). The characteristics of the patients are listed in Table 1. The expression of KLF8 in gastric cancer was also analysed in TCGA Illumina RNA‐Seq datasets. Gene‐level expression data and clinical characteristics of gastric cancer samples including patient clinical pathological parameters and KLF expression levels were downloaded from the TCGA database (https://genome-cancer.ucsc.edu/). Criteria for inclusion were as follows: (a) pathological diagnosis of adenocarcinoma; (b) patients did not receive systemic chemotherapy prior to surgery; and (c) intact survival information.

**Table 1:** Association between KLF8 expression and clinicopathological factors in gastric cancer patients

Characteristics	Total	KLF8 expression		*P* value
		Low expression	High expression	
Gender				0.522
Male	108	41 (55.4)	67 (50.8)	
Female	98	33 (44.6)	65 (49.2)	
Age				0.420
<60	112	43 (58.1)	69 (52.3)	
≥60	94	31 (41.9)	63 (47.7)	
Histologic grade				0.156
G1/G2	89	37 (50.5)	52 (39.4)	
G3	117	37 (50.0)	80 (60.6)	
Tumour diameter				**<0.001**
<5 cm	102	53 (71.6)	49 (37.1)	
≥5 cm	104	21 (28.4)	83 (62.9)	
T stage				**0.003**
T1	3	3 (4.1)	0 (0)	
T2	34	17 (23.0)	17 (12.9)	
T3	99	38 (51.4)	61 (46.2)	
T4	70	16 (21.6)	54 (40.9)	
N stage				**<0.001**
N0	61	28 (37.8)	33 (25.0)	
N1	51	27 (36.5)	24 (18.2)	
N2	56	19 (25.7)	37 (28.0)	
N3	38	0 (0)	38 (28.8)	
Lymphovascular invasion				0.091
Negative	159	62 (83.8)	97 (73.5)	
Positive	47	12 (16.2)	35 (26.5)	
Perineural invasion				0.119
Negative	185	56 (75.7)	97 (73.5)	
Positive	67	18 (24.3)	35 (26.5)	

Bold type indicates statistical significance

This study was approved by Minhang Hospital, Fudan University Research Ethics Committee. Informed consent was obtained from each enrolled patient.

### 2.2 RNA extraction, reverse transcription and qRT‐PCR analysis

Total RNA extraction, reverse transcription and qRT‐PCR analysis were performed as previously described.^30^ The primers for qRT‐PCR analysis were synthesized by Huagene (Shanghai, PRC). The relative expressions of target genes were calculated and normalized using the RQ value method relative to β‐actin.

### 2.3 IHC staining

Staining was performed as previously described.^30^ Commercial monoclonal antibodies to KLF8 and GLUT4 were used as primary antibodies and PBS was used as a negative control. Evaluation of the results was performed according the criteria as recommended by the manufacturer.

IHC scores were determined according to positively stained cells and staining intensity. Positive staining was scored as 0 (<5%), 1 (5%‐25%), 2 (26%‐50%), 3 (51%‐75%) and 4 (>75%) according to the percentages of the positive staining areas in relation to the whole carcinoma area. The staining intensity was scored as 0 (negative), 1 (weak), 2 (medium) or 3 (strong). IHC total scores were calculated as a product of the positive staining score multiplied by the stain intensity score.^30^ Samples with a final staining score of ≤4 were considered low expression and those with a score of >4 were considered high expression.

### 2.4 Western blot

Western blotting assay was performed as previously described.^31^ Briefly, total proteins were separated by SDS‐PAGE and transferred onto PVDF membranes (Bio‐rad). Membranes were detected with primary antibodies overnight. Membranes were then incubated with corresponding secondary antibodies and visualized using enhanced chemoluminescence (Pierce, Thermo Scientific).

### 2.5 Cell culture

Human gastric cancer cell lines AGS and MGC803 were used for in vitro study. The cells were grown in RPMI‐1640 medium (Gibco) containing 10% foetal bovine serum in a humidified 37ºC incubator supplemented with 5% CO_2_.

### 2.6 Plasmids and the establishment of stable cell lines

Short hairpin RNA (shRNA) targeting the KLF8 coding sequence (5′‐CAGCACTGTTTAATGACAT‐3′) was inserted into pLKO.1 plasmid. Scramble sequence (5′‐TTCTCCGAACGTGTCACGT‐3′) was used as negative control. AGS and MGC803 cells were transfected with the pLKO.1‐shKLF8 expression vector or pLKO.1‐scramble Transfected cells were selected using puromycin to obtain stable KLF8 knockdown and control cells.

### 2.7 Immunofluorescence

Immunofluorescence was performed as previously described.^29^ Briefly, cells were fixed in 4% paraformaldehyde for 20 minutes, incubated in blocking buffer, and then incubated with GLUT4 antibody followed by Alexa Flour 594 TgG donkey anti‐rabbit (Invitrogen). DAPI was used to detect nuclei.

### 2.8 Glycolysis analysis

Glucose consumption and lactate production were detected using the Glucose Uptake Colorimetric Assay Kit (Biovision, Milpitas, CA) and Lactate Colorimetric Assay Kit (Biovision), respectively, according to the manufacturer's protocols. RT‐PCR was performed to check the expression levels of glycolytic enzymes. All reactions were run in triplicate.

### 2.9 Luciferase assays

The GLUT4 promoter (from +100 to −2000 bp) was cloned into the pGL3 basic vector (Promega, Madison, WI, USA) to produce pGL3‐GLUT4‐Luc. MGC803 and AGS cells cultured in 96‐well plates were cotransfected with the pGL3‐GLUT4‐Luc or pGL3 vector along with pcDNA3.1‐KLF8/pcDNA‐control and Renilla plasmid. After 48 hours, cells were lysed and examined using a dual luciferase assay kit (Promega) according to the manufacturer's protocol. All experiments were done in triplicate. Data are shown as mean ± standard deviation (SD).

### 2.10 Statistical analysis

Statistical analysis was performed using spss 21.0 statistical package (SPSS, Chicago, IL). Based on requirements, either the *χ* ^2^ or Fisher exacts test was used to investigate the relationships between KLF8 expression and histopathological factors. The 5‐year overall survival (OS) and disease‐free survival (DFS) rate were estimated by the Kaplan‐Meier method. The difference in survival between the groups at each observed event time was compared by the log‐rank test. X‐title program 32 was used to select optimal cut‐off for KLF8 and GLUT4 in the TCGA database. The glycolysis study results were analysed by one‐way analysis of variance or independent sample *t* test. A *P* < 0.05 was considered statistically significant.

## 3 RESULTS

### 3.1 KLF8 is a potential biomarker for gastric cancer

**Figure 1:**
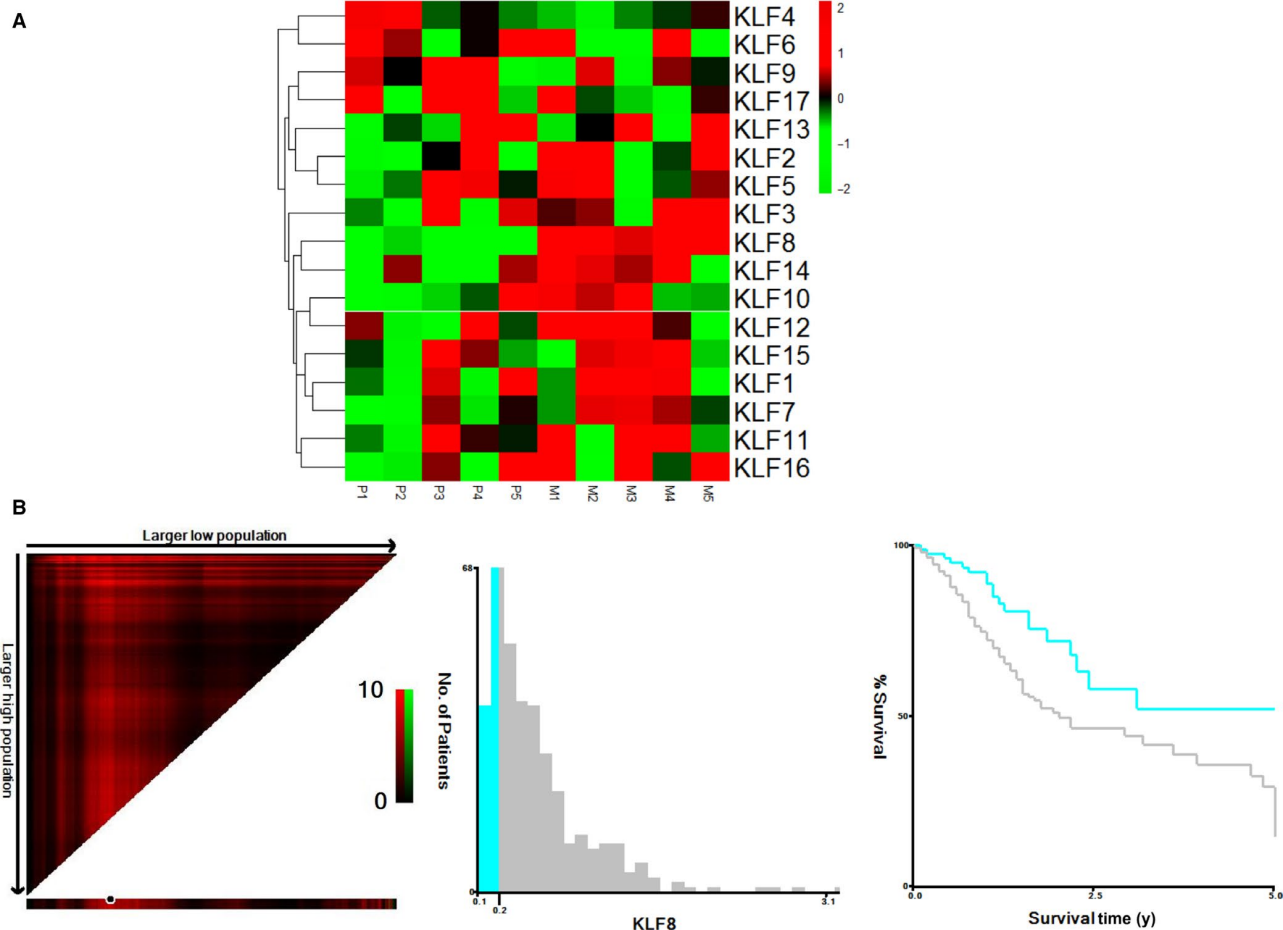
KLF8 is a potential biomarker for gastric cancer. A, Heatmap of KLF family genes in five paired primary gastric cancer tissues and liver metastasis using RT‐PCR. Results were normalized by Z‐score. Each column represents a specimen denoted on the above and each role represents a gene which is denoted on the right. Red colour indicates up‐regulated genes and green colour indicates down‐regulated genes. P: primary gastric cancer tissue; M: corresponding liver metastasis. KLF8 was significantly up‐regulated in liver metastases than corresponding primary tumours. B, *X*‐tile analyses of 5 years overall survival were performed using patient data from TCGA to determine the optimal cut‐off values for KLF8 mRNA. The gastric cancer patients were divided into training and validation sets. X‐tile plots of training sets are shown in the left panels, with plots of matched validation sets shown in the smaller inset. The optimal cut‐off values highlighted by the black circles in left panels are shown in histograms of the entire cohort (middle panels), and Kaplan‐Meier plots are displayed in right panels. *P* values were determined using the cut‐off values defined in training sets and applying them to validation sets. High KLF8 expression was associated with shorter overall survival (*P* = 0.006)

To identify KLF family members that may be involved in gastric cancer metastasis, we performed RT‐PCR to evaluate the gene expression levels of KLF family members in five paired liver metastases and corresponding primary tumours from gastric cancer patients diagnosed with synchronous liver metastasis. The results showed that KLF8 expression was the most significantly up‐regulated among KLF members in liver metastases compared with primary tumours (Figure 1A).

We next investigated the prognostic value of KLF8 in the TCGA database. KLF8 mRNA expression level was first treated as continuous variable, and it was validated as a prognostic biomarker in univariate analysis (HR: 1.500, 95% CI 1.021‐2.204, *P* = 0.039). We used the X‐title program to divide patients into high and low KLF8 expression groups and found that the 5 years OS was 26.6% in patients with high KLF8 expression compared with 50.8% in patients with low KLF8 expression (*P* = 0.006) (Figure 1B).

### 3.2 Clinical significance of KLF8 in a validation cohort

**Figure 2:**
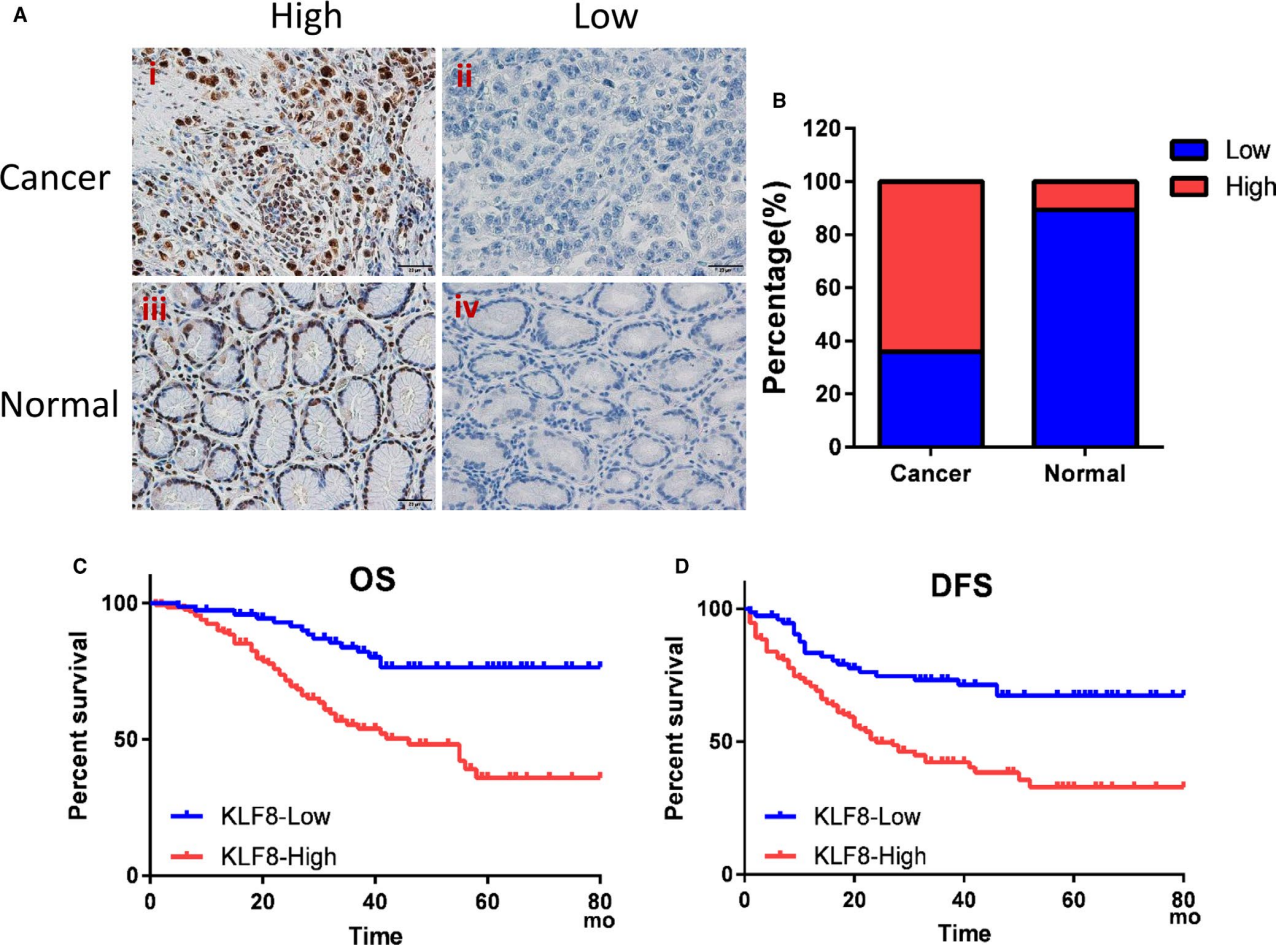
Overexpression of KLF8 is correlated with poor survival for gastric cancer patients. KLF8 expression was detected by immunohistochemical staining in gastric cancer tissues. KLF8 was mainly localized in the nuclei of tumour cells. A, Examples of high (i) or low (ii) KLF8 expression in gastric cancer, and high (iii) or low (iv) KLF8 expression in normal gastric tissues. Magnification, 400×. B, The percentage of high KLF8 expression was significantly higher in gastric cancer than normal controls (*P* = 0.001). (C, D) Patients were divided into KLF8 high and low expression groups and Kaplan‐Meier analyses were performed to evaluate the correlation of KLF8 expression and survival. (C) Overexpression of KLF8 was correlated with worse OS (*χ* ^2^ = 19.15, *P* < 0.001). (D) Overexpression of KLF8 was correlated with worse DFS (*χ* ^2^ = 17.58, *P* < 0.001)

As the TCGA database lacks therapy information, we then validated the significance of KLF8 by IHC in gastric cancer using our own database of 206 gastric cancer samples. Representative images of KLF8 IHC staining are shown in Figure 2A. KLF8 was highly expressed in 64.1% of cancer tissues compared with 10.68% in normal controls, and the difference was statistically significant (Figure 2B). Chi‐square analysis indicated that high KLF8 expression significantly correlated with larger tumour diameter (*P* < 0.001), advanced T stage (*P* = 0.003) and N stage (*P* < 0.001), suggesting that KLF8 may promote tumour progression (Table 1).

Of the 206 patients, 96 patients (46.6%) showed tumour recurrence during the median follow‐up period of 35 months (range, 1‐82 months) and 69 patients (33.5%) died of the disease. The recurrence rate was 56.06% (74/132) and 29.73% (22/74) in patients in the KLF8 high and low expression groups, respectively. High KLF8 expression was significantly correlated with worse DFS for patients with gastric cancer after gastrectomy (*χ* ^2^ = 17.58, *P* < 0.001; Figure 2C). The 5 years OS was 35.6% and 76.3% for patients in high and low KLF8 expression groups, respectively (*χ* ^2^ = 19.15, *P* < 0.001) (Figure 2D). These results suggested that KLF8 could promote tumour growth and metastases, thus contributing to poor prognosis in gastric cancer.

### 3.3 KLF8 promotes glycolysis in gastric cancer

**Figure 3:**
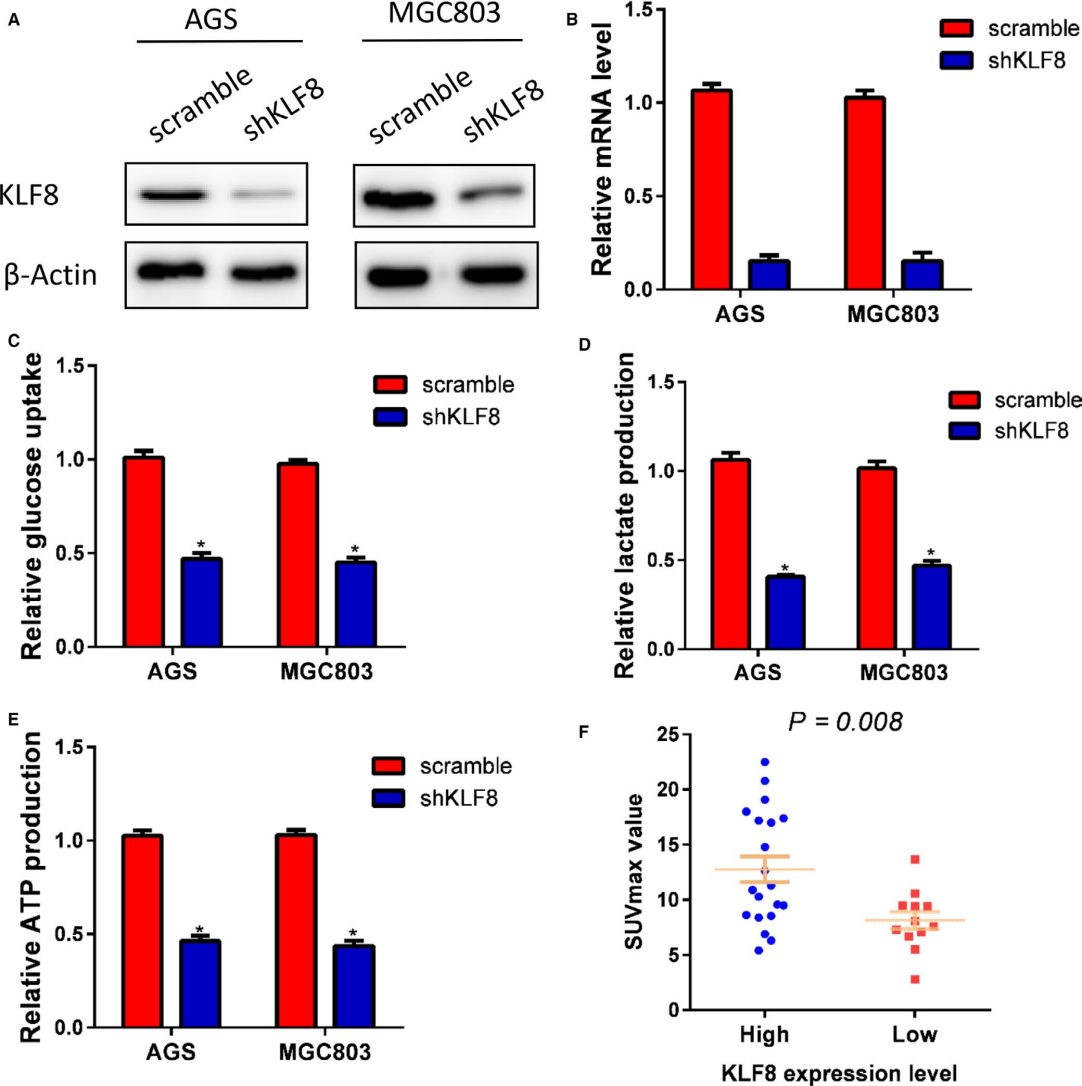
KLF8 promotes glycolysis in gastric cancer. KLF8 expression was silenced by shRNA in AGS and MGC803 cells, and knockdown efficacy was confirmed by Western blotting (A) and RT‐PCR (B). Knockdown of KLF8 expression significantly decreased glucose consumption (C), lactate production (D) and ATP production (E). Patients with high KLF8 expression exhibited higher SUVmax value than those with low KLF8 expression (*P* = 0.008). *P < 0.05

Tumour growth and metastases require glucose metabolism reprogramming to glycolysis. We thus next investigated whether KLF8 may play a function in glycolysis in gastric cancer. KLF8 expression was silenced in AGS and MGC803 gastric cancer cells by lentivirus‐mediated method, and knockdown efficiency was confirmed by RT‐PCR and Western blotting (Figure 3A,B). We then calculated the glucose utilization, lactate concentrations and ATP production in the gastric cancer cells. Knockdown of KLF8 expression significantly decreased the glucose utilization, lactate concentrations, and ATP production in both MGC803 and AGS cells (Figure 3C‐E). SUVmax in PET/CT scan is a reflection of the Warburg effect rate. In a series of 32 gastric cancer patients, we found higher SUVmax value in patients with high KLF8 expression than those with low expression (Figure 3F).

### 3.4 KLF8 promotes glycolysis by targeting glucose transporter GLUT4

**Figure 4:**
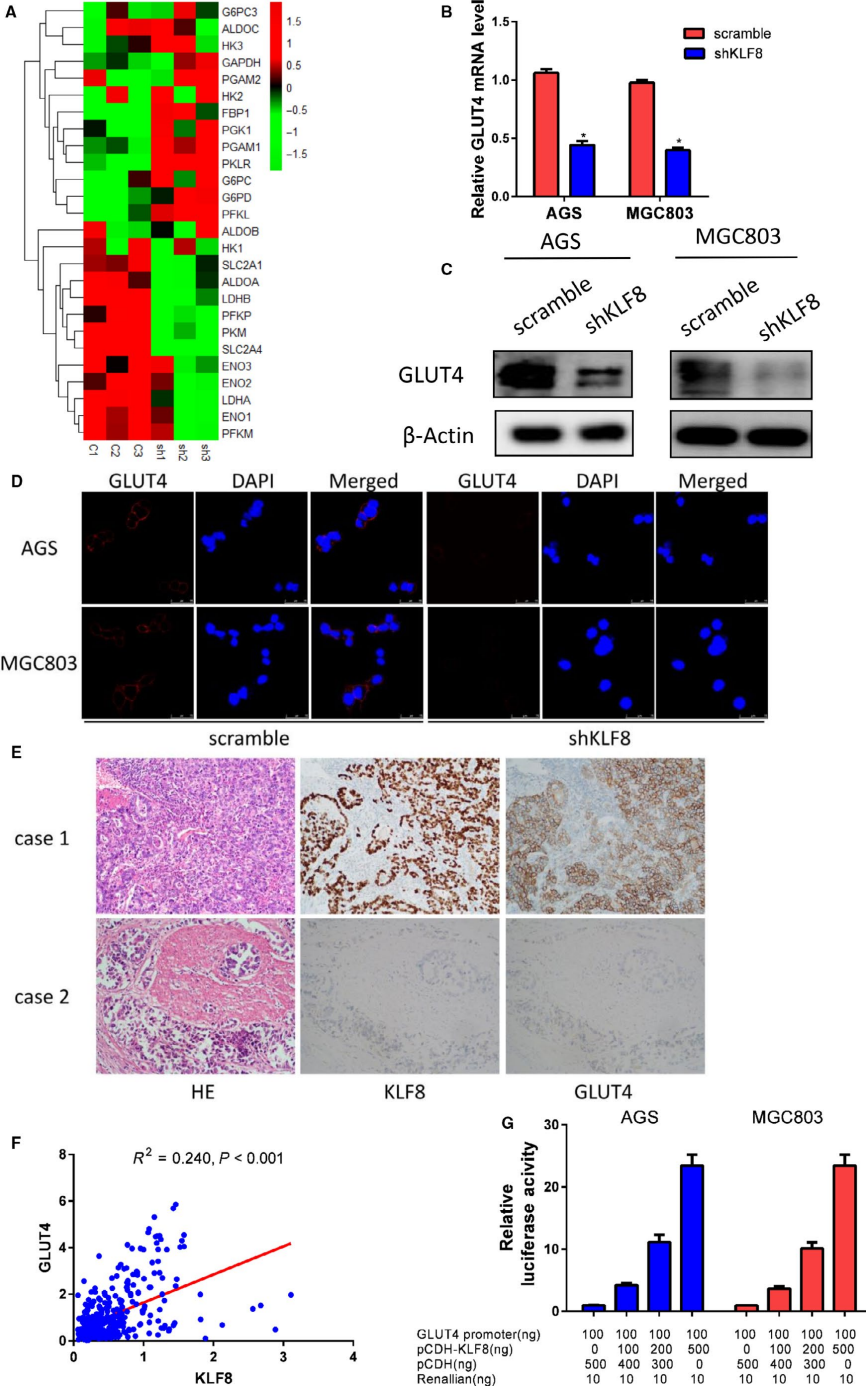
KLF8 promotes glycolysis by targeting glucose transporter GLUT4. (A) Silencing KLF8 up‐regulated or down‐regulated several rate‐limiting genes in glycolysis process, and the most significantly decreased was GLUT4 in AGS cells. sh = shKLF8, c: control. 1, 2, 3 means repeated three times. (B) RT‐PCR and (C) Western blot analysis confirmed that knockdown of KLF8 expression decreased GLUT4 expression in both transcriptional and protein levels in AGS and MGC803 cells. (D) Silencing KLF8 expression decreased GLUT4 expression on cell membranes. (E) KLF8 and GLUT4 show consistent expression patterns in gastric cancer tissues. High KLF8 expression is often accompanied by high GLUT4 expression, and GLUT4 is always expressed at low levels when KLF8 is expressed at low levels. (F) KLF8 was positively correlated with GLUT4 expression in the TCGA database (*P* < 0.001). (G) Dual luciferase assay indicated that KLF8 activated the GLUT4 promoter activity in a dose‐dependent manner. *P < 0.05

Glycolysis is a multi‐step reaction that is regulated by a series of rate‐limiting glycolytic enzymes and glucose transporters. We next investigated the effect of KLF8 on the expression levels of the genes encoding the glycolytic enzymes in AGS cells using RT‐PCR. As shown in Figure 4A, silencing KLF8 resulted in up‐regulation or down‐regulation of several genes encoding glycolytic enzymes, and the most significantly decreased was GLUT4 mRNA in AGS cells. RT‐PCR and Western blot analysis further confirmed that knockdown of KLF8 expression decreased GLUT4 expression at both transcriptional and protein levels in MGC803 and AGS cells (Figure 4B,C). Immunofluorescence showed that knockdown of KLF8 significantly decreased GLUT4 expression on cell membranes (Figure 4D). Importantly, KLF8 and GLUT4 showed similar expression patterns in gastric cancer tissues. High KLF8 expression was often accompanied by high GLUT4 expression, and GLUT4 generally showed low expression when KLF8 was expressed at low levels (Figure 4E). Moreover, there was a positive correlation between KLF8 and GLUT4 transcriptional levels in the TCGA database (*P* < 0.001) (Figure 4F).

To examined whether the KLF8 transcription factor regulated GLUT4, we then constructed a luciferase reporter driven by the human GLUT4 promoter and tested the effect of KLF8 on GLUT4 promoter activity. Dual luciferase assay showed that expression of KLF8 activated GLUT4 promoter activity in a dose‐dependent manner (Figure 4G).

### 3.5 GLUT4 promotes glycolysis and is a biomarker for gastric cancer

**Figure 5:**
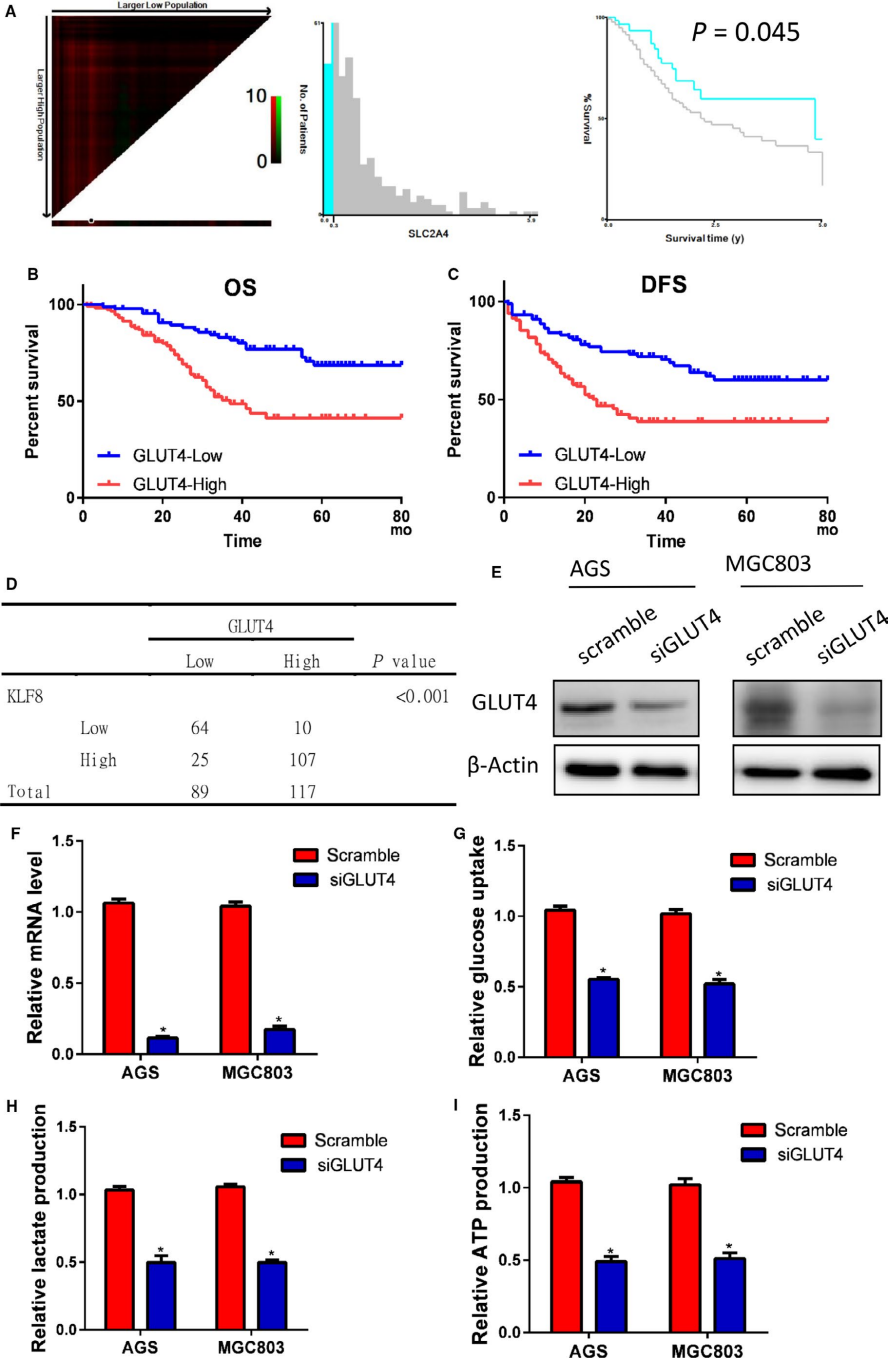
GLUT4 expression in gastric cancer. (A) *X*‐tile analysed indicated that high GLUT4 expression predicted poor survival in gastric cancer (*P* = 0.045). GLUT4 expression was further analysed in the validation cohort using IHC staining. The results demonstrated that patients with higher GLUT4 expression levels had worse OS (B) and DFS (C) rates than did patients with lower GLUT4 expression levels. (D) A positive relationship between GLUT4 and KLF8 expression was also observed in the validation cohort (*P* < 0.001). GLUT4 was knocked down in AGS and MGC803 cells, and the knockdown effect was determined by Western blot (E) and RT‐PCR (F) analysis. Silencing GLUT4 markedly inhibited glucose consumption (G), lactate production (H) and ATP production (I) in both AGS and MGC803 cells (*P* < 0.05). *P < 0.05

We further examined GLUT4 expression in the TCGA database. X‐tile analysis indicated that high GLUT4 expression predicted poor survival in gastric cancer (*P* = 0.045) (Figure 5A). We analysed GLUT4 expression in the validation cohort using IHC staining and survival rates. Patients with higher GLUT4 expression levels had worse OS (Figure 5B) and DFS (Figure 5C) than patients with lower GLUT4 expression levels. A positive relationship between GULT4 and KLF8 expression was also observed in the validation cohort (*P* < 0.001) (Figure 5D).

Finally, we examined the role of GLUT4 in AGS and MGC803 cells using siRNA and first confirmed successful knockdown by Western blot (Figure 5E) and RT‐PCR (Figure 5F) analysis. As demonstrated in Figure 5G‐I, silencing GLUT4 markedly inhibited glucose consumption, lactate production, and ATP production in both AGS and MGC803 cells. 

## 4 DISCUSSION

Gastric cancer is an aggressive malignancy with poor prognosis. Currently, the only potentially curative treatment for gastric cancer is surgery. However, a high percentage of patients show tumour recurrence after surgery. Understanding the prognostic factors or the risk for metastasis is intriguing and could affect clinical practice.^29^

KLF8 is a GT‐box (CACCC) binding dual‐transcription factor^33^ that is widely expressed in adult tissues. KLF8 is aberrantly expressed in several types of human tumours and its high expression is significantly correlated with oncogenic transformation and tumour progression. In the present study, we first systematically examined the gene expressions of KLF family members in liver metastases and corresponding gastric cancer tissues and found that KLF8 was most significantly up‐regulated in liver metastases among all KLF family members. We also demonstrated that KLF8 was a predictor for OS in the TCGA database. Furthermore, we found that KLF8 expression was significantly associated with larger tumour size and advanced TN stage for gastric cancer after gastrectomy in both TCGA and our database.

Unlike differentiated cells, tumour cells have unrestricted potential for division and proliferation. The relentless biosynthesis of macromolecules that are needed for the growth of newly divided cells requires the uptake of glucose and other carbon sources in excess of energetic needs.^30^, ^34^, ^35^ The vast majority of tumour cell types display modified energetic and anabolic metabolism pathways and rates compared with their tissue of origin.^36^ The Warburg effect, also known as aerobic glycolysis, is the conversion from oxidative phosphorylation to glycolysis, which is characterized as increased lactate production even with sufficient oxygen, and is regarded as a hallmark for cancer development and progression.^28^, ^30^, ^37^ Because KLF8 expression correlated with larger tumour size and advanced TN stage in gastric cancer, and as previous studies showed that KLF8 could induce EMT, we considered whether the impact of KLF8 was caused by glucose metabolism transformation. Consistent with this hypothesis, silencing KLF8 expression significantly reduced glucose uptake, lactate secretion and ATP production. Thus, the altered metabolism induced by KLF8 may be essential for cancer cell proliferation and metastasis.

Glycolysis is a multi‐step reaction regulated by a series of glycolytic enzymes, and KLF8 is a classic transcriptional factor. We thus investigated relationship between KLF8 and the expressions of glycolytic enzyme‐related genes. The results showed that KLF8 was closely correlated with GLUT4 expression in both transcriptional and protein levels. Moreover, KLF8 activated the GLUT4 promoter in a dose‐dependent manner. Furthermore, our functional study of GLUT4 indicated that its expression correlated with poor survival of gastric cancer and glycolysis rate in gastric cancer cells, which is consistent with the survival and functional study of KLF8 in gastric cancer. Collectively, these results suggested that GLUT4 may be a potential transcriptional target of KLF8. Previous studies also showed that KLF15 is a transcriptional regulator of GLUT4 in skeletal muscle cells.^38^, ^39^

Our study had several limitations. First, we did not perform animal studies to examine the effects of KLF8 on tumour progression and metastases in vivo, and thus further in vivo studies are needed to validate our findings. Second, the survival of gastric cancer is affected by other factors, such as medical insurance status, radical surgical resection, and neoadjuvant or adjunct therapy, and thus biomarkers of a single gene are not sufficient. Third, although we confirmed a transcriptional relationship between KLF8 and GLUT4 with multiple data, chromatin immunoprecipitation assay is required to confirm KLF8 direct binding to the GLUT4 promoter.

In conclusion, here we demonstrate that KLF8 is a novel prognostic biomarker for survival in gastric cancer, and silencing KLF8 expression impairs glycolysis by targeting GLUT4 in gastric cancer cells. Hence, KLF8 may be a new biomarker and potential therapeutic target for gastric cancer treatment.

## CONFLICT OF INTEREST

The authors confirm that there is no conflict of interest.
